# Prenatal diagnosis, ultrasound findings, pregnancy outcomes and follow-up information analysis of xp22.31 deletion /duplication syndrome

**DOI:** 10.1186/s12884-026-09837-0

**Published:** 2026-08-19

**Authors:** Xiaojin Luo, Yuanyuan Pei, Xiaohang Chen, Jinmao Xu, Hongyan Niu, Fei Zhou, Shuxian Zeng, Shuangwu Li, Jinxing Liu, Weiqiang Liu, Fengxiang Wei

**Affiliations:** 1 The Genetics Laboratory, Longgang District Maternity & Child Healthcare Hospital of Shenzhen City (Longgang Maternity and Child Institute of Shantou University Medical College), Shenzhen, Guangdong China; 2 The Ultrasonography Department, Longgang District Matemity & Child Healthcare Hospital of Shenzhen city ( Longgang Matemity and child Institute of Shantou University Medical College ), Shenzhen, Guangdong China

**Keywords:** Prenatal diagnosis, Xp22.31 deletion, Xp22.31 duplication, SNP-array, Ultrasound findings

## Abstract

**Objective:**

Analyze the ultrasound features, single nucleotide polymorphism microarray (SNP-array) outcomes, pregnancy results, and follow-up data of fetuses with Xp22.31 deletion or duplication in mid to late pregnancy.

**Methods:**

A retrospective analysis was carried out on 10 cases diagnosed with Xp22.31 deletion and 11 cases with duplication, identified through SNP-array testing. The analysis focused on assessing ultrasound abnormalities, tracing parental origins, pregnancy outcomes, and postnatal follow-up conditions.

**Results:**

The deletion sizes in the 10 deletion cases ranged from 913 Kb to 1.81 Mb, while the duplication sizes in the 11 duplication cases ranged from 1.29 Mb to 2.35 Mb, encompassing nine OMIM genes including STS, ANOS1, DXS1283E, VCS3B, and HDHD1A. Among the fetuses with deletions, four cases (40.0%, 4/10) exhibited ultrasound abnormalities, which included cardiovascular system malformations, lateral ventriculomegaly, clubfoot, increased nuchal translucency, and polyhydramnios. Notably, Case 5 involved a fetus with complex multi-organ malformations, including bilateral ventriculomegaly, a ventricular septal defect, and clubfoot. Among the 11 cases of duplications, three cases (27.3%, 3/11) exhibited ultrasound abnormalities, primarily featuring cardiovascular malformations, ventriculomegaly, and polyhydramnios. Of the 10 deletion cases, eight underwent parental origin testing, with seven cases inherited (six maternal and one paternal) and one case of *de novo* mutation. Among the 11 duplication cases, eight underwent parental origin testing, with seven cases being inherited (four maternal and three paternal) and one case representing a *de novo* mutation. Of the 10 deletion cases, six chose to terminate the pregnancy, while four decided to continue. Follow-up revealed that Case 6 was diagnosed with STS ichthyosis after birth, and the sbling of Case 3 and Case 10 exhibited delayed walking and speech development. In the duplication group, one case opted for termination of pregnancy, while 10 continued with their pregnancies. Follow-up identified that Case 18 exhibited delayed walking and speech development.

**Conclusion:**

This study provides a preliminary assessment of the genotypes and phenotypes of fetuses with Xp22.31 deletion /duplication, expanding the phenotypic spectrum of Xp22.31 deletion /duplication syndrome. The findings indicate that prenatal Xp22.31 deletion in male fetuses are likely to manifest as STS ichthyosis after birth. Xp22.31 deletion and duplication may be associated with cardiovascular malformations. For fetuses with Xp22.31 deletion /duplication that appear phenotypically normal prenatally, postnatal monitoring of their neurological development is crucial.

## Introduction

Prenatal screening estimate that Xp22.31 deletion occur in approximately 1 in 1500 males and 1 in 750 females [1]. Among males carrying this variant, 25% to 60% are diagnosed with X-linked ichthyosis (MIM:308100), a skin disorder characterized by skin scaling, caused by a deficiency of the steroid sulfatase (STS) enzyme [2]. In male patients, XLI deletions typically ranging from 1.5 to 1.7 Mb are associated with extracutaneous symptoms, including corneal opacity, testicular dysgenesis, focal epilepsy, atopic diseases, abnormal coagulation and fibrosis, as well as an increase in neurodevelopmental and emotional characteristics [3]. Larger deletions (> 2.5 Mb) may be associated with more severe developmental issues [4]. Report indicated that the frequency of Xp22.31 duplication can be as high as 0.41% in the general population [5]. However, the pathogenicity of Xp22.31 duplication is controversial. Some studies suggest that these duplications represent variants of uncertain significance (VUS). Others interpret Xp22.31 duplications as benign, while some research suspects they might be pathogenic. Associations include neurodevelopmental changes, intellectual disability, cognitive deficits, and epileptic seizures, but the pathogenicity and expressivity remain under debate [6]. Therefore, interpretations vary among different diagnostic laboratories, causing significant confusion for clinical genetic counseling. This study, through retrospective analysis of ultrasound findings, SNP-array results, pregnancy outcomes, and follow-up of 21 cases with Xp22.31 deletion /duplication, further enriches and supplements the clinical phenotype spectrum of Xp22.31 deletion /duplication in mid to late pregnancy.

## Subjects and methods

### Subjects

Xp22.31 deletion and duplication were identified through amniocentesis and SNP-array testing at the Prenatal Diagnosis Center of Longgang District Maternal and Child Health Hospital in Shenzhen, from September 2017 to March 2025. The analysis focused on ultrasound abnormalities, SNP-array results, parental origins, pregnancy outcomes, and postnatal follow-up conditions. The primary indications for prenatal diagnosis in these pregnant women included fetal ultrasound abnormalities, high-risk NIPT screening results, increased nuchal translucency, lateral ventriculomegaly, advanced maternal age, and adverse pregnancy history. Before undergoing invasive prenatal diagnosis and SNP-array testing, all participants were fully informed about the study’s purpose, procedures, and potential risks, and signed informed consent forms. When choosing to continue the pregnancy, follow-up phone calls were made to track the newborns’ growth and development. This study exclusively included cases of isolated Xp22.31 deletion /duplication, excluding cases involving other chromosomal variations. The study was approved by the Ethics Review Committee of Longgang District Maternal and Child Health Hospital (No. LGFYYXLL-028).

### Method

#### SNP-array

Collect 10 mL of amniotic fluid or 2 mL of EDTA anticoagulated blood, and extract DNA using the QIAamp DNA Kit (Qiagen, Germany). Measure the genomic DNA concentration with an ultramicro spectrophotometer. Purify using magnetic beads to remove excess enzymes and primers, obtaining purified polymerase chain reaction (PCR) amplified products. Further fragment the genomic DNA and label it using a terminal deoxynucleotidyl transferase. Place the labeled products into the Affymetrix Cytoscan 750 K Array chip (Thermo Fisher Scientific, USA) and hybridize for 18 h. The following day, wash and stain to remove unhybridized DNA, and finally, read the data using a chip scanner.

#### Data analysis

The results obtained are compared with the Database of Genomic Variants (http://dgv.tcag.ca/) to exclude common polymorphic copy number variations (CNVs). The remaining CNVs are further investigated using databases such as DECIPHER (http://decipher.sanger.ac.uk/), ISCA (http://dbsearch.clinicalgenome.org/search/), ClinGen (http://www.ncbi.nlm.nih.gov/projects/dbvar/clingen/), ClinVar (http://www.ncbi.nlm.nih.gov/clinvar/), OMIM (http://omim.org/), and PubMed (http://pubmed.ncbi.nlm.nih.gov/). According to the guidelines of the American College of Medical Genetics and Genomics (ACMG), the clinical significance of these CNVs is assessed to determine their pathogenicity.

#### Follow-up

Follow-ups were conducted within two weeks of the pregnant woman’s expected delivery date, documenting fetal ultrasound abnormalities, pregnancy outcomes, mode of delivery, and the phenotypes of carrier parents after parental origin was confirmed. Once the child’s birth was confirmed, regular follow-ups were carried out at 6, 12 and 24 months postnatally (with extended follow-up for children who had abnormal prenatal genetic test results). At these time points, the Gesell Developmental Diagnostic Scale was used to assess the children’s neurodevelopmental status, while their postnatal growth and development were also recorded. The latest follow-up was completed on September 15, 2025. Among the children under follow-up, the oldest was 6 years and 5 months old, and the youngest was 18 months old.

## Results

### Analysis of SNP-array results in 21 cases with Xp22.31 deletion /duplication

**Fig. 1:**
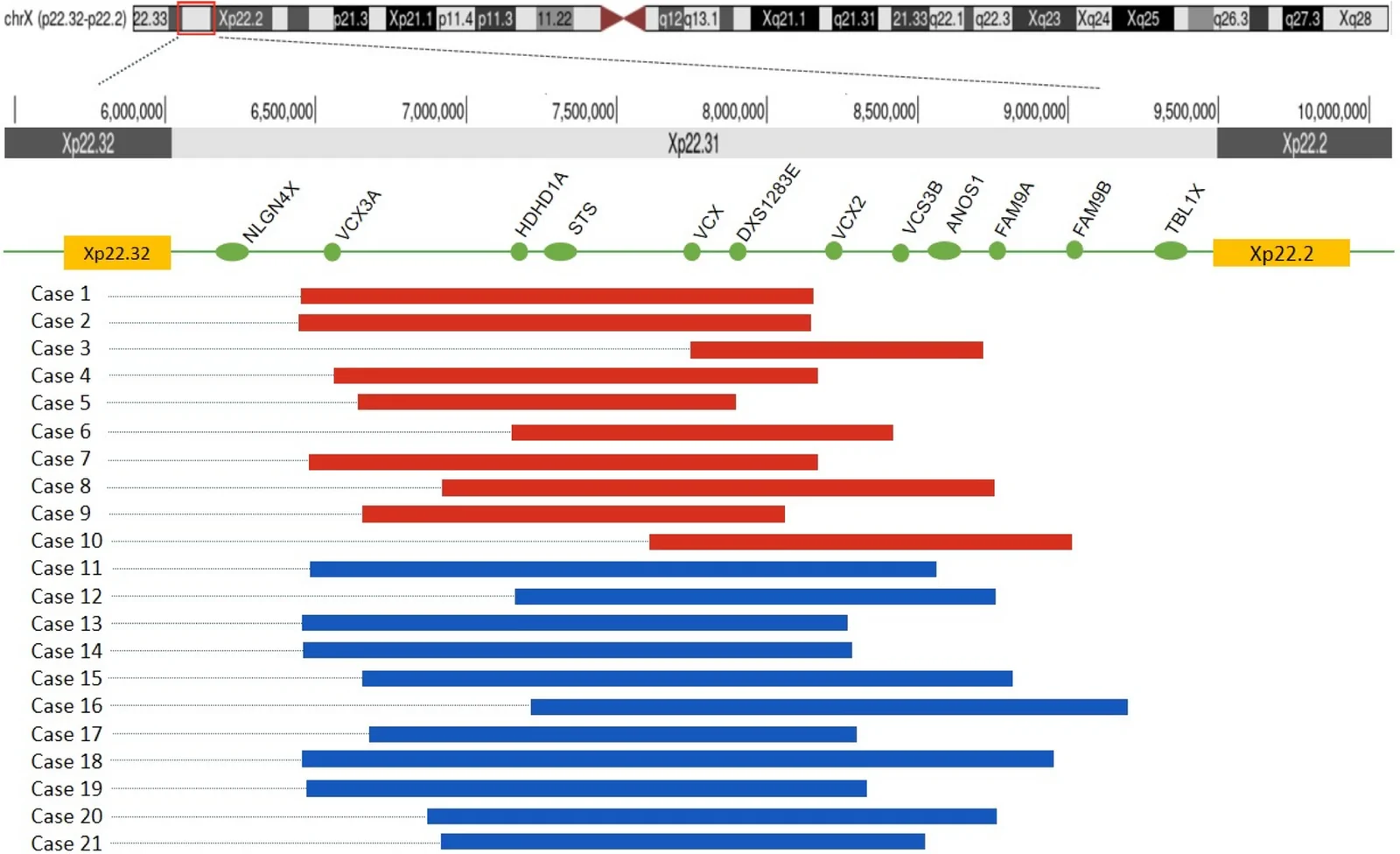
The schematic diagram illustrates the locations, sizes, and the OMIM genes contained within the Xp22.31 deletions and duplications for the 21 cases. Cases 1 to 10 represent the deletion cases, and cases 11 to 21 represent the duplication cases

The average age of the 21 pregnant women with Xp22.31 deletions and duplications was 29.3 ± 4.69 years (range, 16–48 years). The mean gestational age was 21.22 ± 2.08 weeks (range, 12–25 weeks). Among these 21 cases, two had a history of adverse pregnancy outcomes, one had gestational hypertension, and none had a history of intrauterine viral infection (Table 1). Among the 21 cases, there were 10 cases of Xp22.31 deletion and 11 cases of duplication. The deletion cases, from Case 1 to Case 10, had deletion sizes ranging from 913Kb to 1.81 Mb. The duplication cases, from Case 11 to Case 21, had duplication sizes ranging from 1.29 Mb to 2.35 Mb. The Xp22.31 deletion /duplication region involved nine OMIM genes including STS, ANOS1, DXS1283E, VCS3B, HDHD1A, and NLGN4X, with STS and ANOS1 identified as haploinsufficient genes. (Fig. 1). Parental origin was verified in eight of the 10 deletion cases, with seven being inherited (six maternal, one paternal) and one de novo mutation. In the 11 duplication cases, parental origin was verified in eight cases, with seven being inherited (four maternal, three paternal) and one de novo mutation. (Table 2).

**Table 1:** Baseline characteristics of 21 cases with Xp22.31 deletions and duplications

Clinical basic characteristics	Number
Maternal age (mean)	24–39 yrs (31.33 ± 4.98)
Gestational weeks	
< 20 weeks	6
20–23 weeks	12
> 23 weeks	3
Adverse pregnancy history	
Yes	2
No	19
Gestational complications	
Gestational hypertension syndrome	1
No complications	20
Intrauterine viral infection	
Yes	0
No	21
Pregnancy risk factors	
High risk of serum screening	7
High risk of NIPT results	5
AMA	5
Adverse pregnancy history	2
Abnormal ultrasound findings	7

**Table 2:** Prenatal indications, SNP-array results, and follow-up information of 18 cases with Xp22.31 deletion and duplication

Case	Age	GW	High risk / Ultrasound findings	SNP-array results	Size	Gender	Inheritance	Outcome	Follow-up information
1	37	19 ^+ 2^	High risk of serum screening (1:140 for T21 ), AMA	arr[hg19]Xp22.31(6,455,151−8,143,319)x0	1.69 Mb	Male	Inherited (maternal)	TOP	N/A
2	38	18	High risk of NIPT ( X chromosome ), AMA	arr[hg19]Xp22.31(6,455,151−8,140,076)x0	1.69 Mb	Male	Inherited (maternal)	TOP	N/A
3	26	23 ^+ 5^	Left LVW (11.4 mm)、polyhydramnios	arr[hg19]Xp22.31(7,815,192−8,728,908)x0	913Kb	Male	Inherited (maternal)	TOP	A daughter speech delays at 3 years and 8 month old
4	31	23 ^+ 2^	Adverse pregnancy history ( child with trisomy 21 )	arr[hg19]Xp22.31(6,598,941−8,128,226)x0	1.53 Mb	Male	Unknown	TOP	N/A
5	21	23	Left LVW(11.2 mm), VSD、strephenopodia	arr[hg19]Xp22.31(6,642,019 − 7,861,665)x1	1.22 Mb	Female	Inherited (maternal)	TOP	N/A
6	32	20 ^+ 5^	High risk of serum screening (1:90 for T21 )	arr[hg19]Xp22.31(7,160,672⁃8,421,663)x1	1.26 Mb	Male	Inherited (paternal)	Live birth	STS diagnosed
7	33	22 ^+ 6^	Increased NT(2.5 mm ), polyhydramnios	arr[hg19]Xp22.31(6,449,559−8,138,771)x1	1.69 Mb	Female	Inherited (maternal)	TOP	N/A
8	33	22	Tricuspid regurgitation, High risk of NIPT ( X chromosome )	arr[hg19]Xp22.31(6,915,192−8,728,928)x1	1.81 Mb	Female	Inherited (maternal)	Live birth	Surgery for cardiac malformation after birth, dry skin
9	30	22	High risk of serum screening (1:150 for T21 )	arr[hg19]Xp22.31(6,689,543−8,081,882)x1	1.39 Mb	Female	Unknown	Live birth	No visible abnormalities
10	39	22	AMA、High risk of serum screening (1:120 for T21 ),	arr[hg19]Xp22.31(7,612,544−8,968,522)x1	1.36 Mb	Female	De novo	Live birth	Speech & motor delays at 3 years and 6 month old
11	24	21 ^+ 3^	Adverse pregnancy history (child with ID)	arr[hg19]Xp22.31(6,488,251−8,474,609)x2	1.99 Mb	Male	Unknown	Live birth	No visible abnormalities
12	35	23	LVW (11.6 mm)	arr[hg19]Xp22.31(7,268,152−8,721,772)x2	1.45 Mb	Male	De novo	Live birth	No visible abnormalities
13	27	25	AS、polyhydramnios	arr[hg19]Xp22.31(6,455,152−8,135,644)x2	1.68 Mb	Male	Inherited (paternal)	TOP	N/A
14	32	21 ^+ 5^	High risk of NIPT ( X chromosome )	arr[hg19]Xp22.31(6,455,152−8,143,319)x3	1.69 Mb	Female	Unknown	Live birth	No visible abnormalities
15	36	20	AMA, High risk of serum screening (1:150 for T21 )	arr[hg19]Xp22.31(6,880,558−8,740,918)x3	1.86 Mb	Female	Inherited (maternal)	Live birth	Speech & motor delays at 3 years and 6 month old
16	26	18 ^+ 3^	High risk of NIPT ( X chromosome )	arr[hg19]Xp22.31(7,240,227−9,142,336)x3	1.90 Mb	Female	Inherited (maternal)	Live birth	Epilepsy
17	29	18	High risk of NIPT ( X chromosome )	arr[hg19]Xp22.31(6,849,233−8,135,644)x3	1.29 Mb	Female	Inherited (paternal)	Live birth	No visible abnormalities
18	33	23	VSD, Increased NT(2.8 mm )	arr[hg19]Xp22.31(6,455,151−8,805,568)x3	2.35 Mb	Female	Inherited (paternal)	Live birth	Surgery for cardiac malformation after birth
19	32	20 ^+ 5^	High risk of serum screening (1:180 for T21 )	arr[hg19]Xp22.31(6,488,251−8,145,904)x3	1.66 Mb	Female	Inherited (maternal)	Live birth	No visible abnormalities
20	26	18	High risk of serum screening (1:110 for T21 )	arr[hg19]Xp22.31(6,958,335−8,695,964)x3	1.74 Mb	Female	Unknown	Live birth	No visible abnormalities
21	38	19 ^+ 4^	AMA	arr[hg19]Xp22.31(7,058,478−8,495,226)x3	1.44 Mb	Female	Inherited (maternal)	Live birth	No visible abnormalities

*Abbreviations: GW* Gestational week, *AMA* Advanced maternal age, *NT* Nuchal translucency, *NIPT* Non-invasive prenatal testing, *LVW* Lateral ventricle widened, *VSD* Ventricular septal defect, *AS* Aortic stenosis, *TOP* Termination of pregnancy, *N/A* Not applicable

### Ultrasound manifestations of fetuses with Xp22.31 deletion

**Fig. 2:**
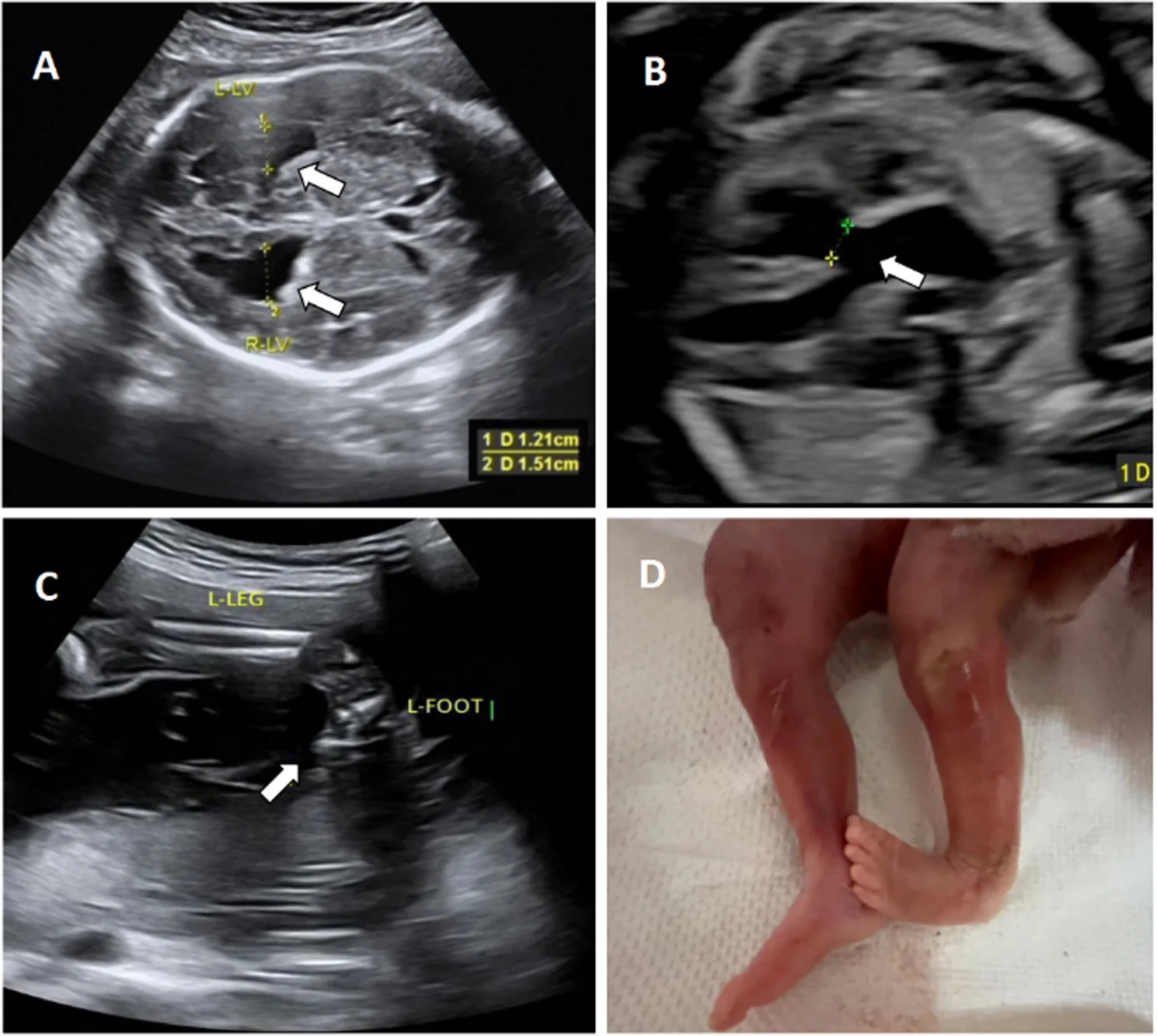
**A** Bilateral lateral ventriculomegaly. **B** Fetal ventricular septal defect. **C** Fetal left talipes equinovarus. **D** Fetal autopsy showing left talipes equinovarus

Among the 10 cases of Xp22.31 deletion, four fetuses (40.0%, 4/10) exhibited ultrasound abnormalities (Table 3). Two of the fetuses had cardiovascular malformations: one (Case 5) had a ventricular septal defect, and another (Case 8) had tricuspid regurgitation. Case 5 involved a female fetus with complex multi-organ malformations, including bilateral lateral ventriculomegaly, ventricular septal defect, and clubfoot (Fig. 2). The deletion segment in this case was inherited from the mother, who has a normal clinical phenotype, a stable personality, and has been working in accounting since graduating from university with a bachelor’s degree. Case 8 involved a female fetus with an Xp22.31 deletion segment also inherited from the mother, who has a normal clinical phenotype. Other detected ultrasound phenotypes included two cases of polyhydramnios, one case of lateral ventriculomegaly, one case of clubfoot, and one case of increased nuchal translucency.

**Table 3:** Ultrasound features of fetuses with Xp22.31 deletion

Ultrasound findings	Cases
Cardiovascular malformation	2
Ventricular septal defect	1
Tricuspid regurgitation	1
Polyhydramnios	2
Strephenopodia	1
Lateral ventricle widened	1
NT thickened	1
Total	7

### Ultrasound findings of fetuses with Xp22.31 duplication

Among the 11 fetuses of duplication, three fetuses (27.3%, 3/11) exhibited ultrasound abnormalities (Table 4). Two of the fetuses had cardiovascular malformations: one (Case 13) had aortic stenosis, and another (Case 18) had a ventricular septal defect. In both cases, the Xp22.31 duplication segment was inherited from the father, who, despite having the same duplication segment, has a normal clinical phenotype. Other detected ultrasound phenotypes included one case of lateral ventriculomegaly, one case of increased nuchal translucency, and one case of polyhydramnios.

**Table 4:** Ultrasound features of fetuses with Xp22.31 duplication

Ultrasound findings	Cases
Cardiovascular malformation	2
Aortic stenosis	1
Ventricular septal defect	1
Lateral ventricle widened	1
Polyhydramnios	1
NT thickened	1
Total	5

### Pregnancy outcomes and follow-up information

Among the 10 cases of Xp22.31 deletion, six chose to terminate the pregnancy. Follow-up revealed that the mother of Case 3 has a daughter with delayed speech and motor development. Of the four cases that chose to continue the pregnancy, follow-up found that Case 6 was diagnosed with STS ichthyosis after birth; this boy’s father also has a similar deletion segment and exhibits a mild ichthyosis phenotype. Case 8, a girl, had prenatal ultrasound findings of tricuspid regurgitation; she had a good prognosis after heart surgery post-birth but exhibited mild dry skin. Case 10 was diagnosed with delayed speech and motor development at the age of 2 years and 7 months, and the Xp22.31 deletion was identified as a de novo mutation (Table 4).

Among the 11 cases of Xp22.31 duplication, one case chose to terminate the pregnancy due to cardiovascular malformations and polyhydramnios. Of the 10 cases that chose to continue the pregnancy, Case 15 was diagnosed with delayed speech and motor development during a physical examination; the mother also possesses the same duplication segment and has a normal clinical phenotype. Case 16 experienced a single epileptic seizure at the age of 3; the mother also has the same duplication segment, has a normal clinical phenotype, and reported no history of seizures. Case 18 was prenatally diagnosed with a ventricular septal defect and underwent surgery at the age of 3, currently with a good prognosis. The mother has the same duplication segment and reported a history of Type II second-degree heart block during past medical examinations (Table 5).

**Table 5:** Seven abnormal phenotypes of Xp22.31 deletions and duplications were found during follow-up and findings from relevant literature

Age at diagnosis	Sibling of Case 3 (deletion)	Case 6 (deletion)	Case 8 (deletion)	Case 10 (deletion)	Case 15 (duplication)	Case 16 (duplication)	Case 18 (duplication)	Study of Esplin et al. (duplications, *n* = 9) [7]
	3 years 8 months	4 years	3 years 6 months	2 years 7 months	3 years 5 months	3 years 2 months	2 years 4 months	15 months−42 years
Gender	Female	Male	Female	Female	Female	Female	Female	N/A
STS / dry skin	No	STS	Dry skin	No	No	No	No	No
Developmental delay	Speech and gross motor delay	No	No	Speech and gross motor delay	Speech and gross motor delay	No	No	33.3% (3/9)
Seizures /epilepsy	No	No	No	No	No	Epilepsy was diagnosed at the age of 3	No	44.4% (4/9)
Cardiovascular malformation	No	No	Tricuspid regurgitation and surgical prognosis is favorable	No	No	No	ventricular septal defect and surgical prognosis is favorable	No

## Discussion

In cases involving duplication and deletion syndrome, the clinical phenotype may not always be apparent before birth, and there is often no clear correlation between prenatal findings and postnatal outcomes [7]. Syndromes that are well-defined in adult patients do not necessarily provide direct evidence for prenatal diagnosis, as prenatal clinical phenotypes cannot reliably be predicted based on the variation’s location and size [8]. The primary mechanisms for CNV formation in the human genome include non-allelic homologous recombination, non-homologous end joining, and microhomology-mediated break-induced replication. Factors such as high repeat sequences and high GC content contribute to chromosomal instability [9]. However, the Xp22.31 region is characterized by notably lower repeat numbers and GC content compared to the average across the human genome, which has sparked scientific interest in the mechanisms underlying CNV occurrence in this area [10]. The Xp22.31 deletion /duplication region involves nine OMIM genes, including STS, ANOS1, DXS1283E, VCS3B, HDHD1A, and NLGN4X, with STS and ANOS1 identified as haploinsufficient genes [11]. Male patients with deletions of the STS gene are clearly associated with X-linked ichthyosis, while female carriers generally do not exhibit the ichthyosis phenotype. Recent studies have found that CNVs in this region may be associated with cardiac and neuropsychiatric abnormalities [12–14]. In this study, ultrasound abnormalities were found in four out of 10 fetuses of Xp22.31 deletion, two of which had cardiovascular malformations, and follow-up identified two cases with neurodevelopmental abnormalities. Among the 11 fetuses of Xp22.31 duplication, three cases showed ultrasound abnormalities, two of which had cardiovascular malformations, and follow-up revealed one case with neurodevelopmental abnormalities. This study suggest that copy number variations in the Xp22.31 region, besides the well-established close association between Xp22.31 deletion and X-linked STS, may also be related to cardiac diseases and neurodevelopmental abnormalities.

The Xp22.31 segment is located in a specific evolutionary region of the X chromosome, characterized by low frequency repeats and lower GC content, involving several functional genes including STS, ANOS1, DXS1283E, VCS3B, HDHD1A, and NLGN4X [15].The STS gene encodes steroid sulfatase, a membrane-bound microsomal enzyme that is expressed and hydrolyzes several 3-hydroxy steroid sulfates [16]. Deficiency of the STS enzyme leads to the accumulation of cholesterol sulfate in the stratum corneum, hindering normal skin desquamation and forming adhesive scales. STS is the pathogenic gene for X-linked ichthyosis [17]. Epidemiological studies have found that boys with X-linked ichthyosis (caused by STS gene deletion) have a higher incidence of attention-deficit/hyperactivity disorder (ADHD). Moreover, a study from 2008 suggested that specific polymorphisms of the STS gene may be associated with susceptibility to ADHD, indicating a potential link with neurological disorders [18]. The ANOS1 gene (Known as KAL1) encodes a protein called Anosmin-1. Anosmin-1 plays a crucial role in embryonic development, particularly in the migration of neurons and the growth of axons. It is located in the extracellular matrix and is involved in regulating the contact and adhesion between neural cells. Mutations in the ANOS1 gene can affect the migration and development of olfactory neurons, leading to Kallmann syndrome or other neurodevelopmental abnormalities [19, 20]. The DXS1283E gene (Known as PNPLA4), expressed in the neural crest cell lineage, is potentially involved in cardiomyocyte differentiation and the formation of cardiac structures. It encodes a protein that possesses triacylglycerol lipase and transacylase activities, contributing to the regulation of lipid metabolism [13, 21]. Increased dosage of the PNPLA4 gene resulting from Xp22.31 duplication is a significant pathogenic risk factor for heterotaxy and congenital heart disease. The pathogenic mechanism involves PNPLA4-mediated regulation of energy metabolism and mitochondrial ATP production, which in turn affects primary cilia formation and function, thereby disrupting embryonic left-right patterning [13]. VCX proteins play a crucial role in proper neurogenesis. Research suggests that deletions within the VCX gene could result in phenotypes associated with intellectual disabilities. Studies have reported that deletions in the Xp22.31 region are associated with the onset of epilepsy. Two children with HDHD1 gene deletions presented with well-controlled focal epilepsy and language developmental delay, leading the study to suggest that HDHD1 is a potential key gene underlying epilepsy caused by Xp22.31 deletions [22]. Labang and colleagues have reported that HDHD1/PUDP and DXS1283E play roles in X-linked intellectual disabilities, as they exhibit high transcription levels in the human brain [23]. Family members with copy number deletions show a potential association with X-linked intellectual disabilities, although the specific mechanisms remain unclear [24]. In this study, Cases 8 and 18 exhibited cardiovascular malformations on ultrasound, while Cases 10, 15, and 16 showed neurodevelopmental abnormalities. This study further suggests that copy number variations in the Xp22.31 region, which includes genes such as ANOS1, DXS1283E, VCX, HDHD1, and STS, may be associated with cardiovascular malformations and neurodevelopmental abnormalities.

Deletion and duplication of Xp22.31 are relatively common in the general population, with deletions considered pathogenic variations that include the STS gene, leading to X-linked ichthyosis (XLI) [25]. The clinical phenotype includes extensive dry, scaly skin and flaking. Nearly all detected patients are male, with symptoms appearing at birth or shortly thereafter. The primary manifestations are widespread scaling on the limbs, face, neck, trunk, and buttocks, with the neck, face, and trunk being the most severely affected [1]. Some patients may also experience complications such as corneal opacity, bronchial asthma, and allergic rhinitis. In females, such cases are rarely reported, although some carriers may show mild scaling and dry skin on the arms and shins [26]. This mild presentation in females can be attributed to the random inactivation of the X chromosome, with a preference for inactivating the structurally abnormal X chromosome [26]. Beyond ichthyosis, benign corneal opacity affects approximately 10% to 50% of male XLI patients, and about 20% have cryptorchidism. Moreover, males with the typical XLI-related deletion (approximately 1.6 Mb) have also been reported to suffer from autism, intellectual disability, epilepsy, developmental delays, and renal abnormalities [27]. Research by Wren and colleagues has demonstrated a strong link between Xp22.31 deletions and arrhythmias, as well as other cardiovascular conditions. As a result, it is advised that individuals diagnosed with Xp22.31 deletions receive cardiac screening promptly to manage and potentially reduce the long-term health consequences associated with arrhythmias [28]. Guidelines from the American College of Medical Genetics and Genomics (ACMG) on the interpretation and reporting of human copy number variations (CNVs) state that the clear association between Xp22.31 deletions and X-linked ichthyosis (XLI) typically categorizes these deletions as pathogenic variants [29].

Xp22.31 duplication is more frequently observed in clinical settings compared to Xp22.31 deletions [12]. The clinical significance is still debated; some consider it a benign variation, others view it as of uncertain significance, and some believe it to be a risk factor [5, 6]. It is considered a benign variation primarily because it is usually inherited from asymptomatic parents [6]. In our study, none of the parents from whom the seven cases inherited the Xp22.31 duplication showed any abnormal clinical phenotype. Li and colleagues conducted tests on 7,793 patients primarily presenting with developmental delays, intellectual disabilities, distinctive facial features, and autism, and found a 0.37% incidence of Xp22.31 duplication, ranging in size from 149Kb to 1.74 Mb. In contrast, the carrier rate in the control group was 0.15% [30]. Further familial testing indicated that the duplication was also present in some of the patients’ mothers and grandmothers, who likewise showed no abnormal characteristics [30]. Nevertheless, various studies have documented a wide range of clinical phenotypes associated with Xp22.31 duplications. Esplin and colleagues documented nine patients with Xp22.31 duplications, who exhibited a range of symptoms such as hypotension, epilepsy, malformed feet, learning difficulties, psychomotor developmental delays, feeding challenges, and autism [12]. Typically inherited, these duplications exhibit significant phenotypic diversity [12]. Consequently, the clinical manifestations associated with Xp22.31 duplications may be influenced by various factors, including the size and integration site of the abnormal X chromosome segment, as well as mechanisms such as random inactivation, skewed inactivation, and gene escape from inactivation [12, 31]. Qiao and colleagues utilized whole-exome sequencing to identify a patient with an Xp22.31 duplication spanning 600 kb (7515787–8123310), which encompassing the VCX and DXS1283E genes. Clinically, this patient presented with hypotension, developmental delays, and physical malformations [32]. This genetic segment was inherited from the mother, who primarily exhibited symptoms of epileptic seizures [32]. In this study, we analyzed 10 fetuses with Xp22.31 deletions, identifying two cases of cardiovascular malformations. Additionally, follow-up assessments revealed two cases with delayed motor and speech development. Among 11 cases with Xp22.31 duplications, we detected two cases of cardiovascular malformations, one case of delayed motor and speech development, and one case of epilepsy. These observations suggest that copy number variations in the Xp22.31 region may be linked to cardiovascular malformations and neurodevelopmental disorders. However, further molecular experimental research is needed to elucidate the underlying mechanisms.

The primary advantage of this study lies in our thorough analysis of the diverse prenatal clinical phenotypes associated with Xp22.31 deletion/duplication syndrome. This has broadened our understanding of the clinical phenotypic spectrum linked to this syndrome and initiated preliminary discussions about the relationship between genotype and phenotype. Nonetheless, there are still some limitations to be addressed. Firstly, due to the diversity in the ages of the newborns and the varying lengths of follow-up, most of the infants are still very young, making it challenging to accurately assess their neuropsychiatric states. Secondly, this study did not thoroughly investigate the correlations between individual gene mutations within the region and clinical phenotypes. Lastly, although the sample size of this study exceeds those of previous studies focusing on prenatal case reports, as a retrospective study, there is still a lack of sufficient data, which may lead to biased conclusions.

## Conclusion

In this study, we further expanded the clinical phenotype spectrum of fetuses with Xp22.31 deletion and duplication at a single prenatal center in a tertiary hospital, and conducted a preliminary evaluation of prenatal ultrasound findings and postnatal clinical phenotypes during follow-up. This study confirmed that the Xp22.31 deletion in male fetuses is closely associated with the phenotype of X-linked ichthyosis, while female fetuses are not affected. It suggests that Xp22.31 deletion and duplication may be closely related to cardiovascular diseases and neurodevelopmental abnormalities. For fetuses with Xp22.31 deletion /duplication that appear phenotypically normal prenatally, it is crucial to closely monitor their neurodevelopment after birth.

## Data Availability

The original data of this study have been submitted to the GEO database(GSE299701).
